# Meetings of Societies

**Published:** 1937

**Authors:** 


### Meetings of Societies

### Bristol Medico-Chirurgical Society

THE seventh meeting of the session was held on Wednesday, 14th April, at 8.30 p.m.

Mr. A. E. Iles was in the Chair, and fifty-two members were present.

Dr. Fraser showed pathological specimens.

Mr. John Wright gave a paper entitled **"The Management of Throat and Nose Infections in Childhood."** Considerable discussion followed, which was contributed to by Mr. Iles, Professor Davy (for Professor Perry), Mr. Hutchinson, Mr. Scarff, Dr. Fraser, Mr. Angell James, Dr. Frank Bodman, and Dr. Gee.

The eighth meeting of the session was held on Wednesday, 5th May, at 8.30 p.m.

Dr. Richard Clarke was in the Chair.

The first Carey Coombs Memorial Lecture was delivered.

The Lecturer, Mr. Laurence O'Shaughnessy, took for his subject **"The Pathology and Surgical Treatment of Cardiac Ischæmia."**

